# *In-vitro* and *In-Vivo* Assessment of 4D Flow MRI Reynolds Stress Mapping for Pulsatile Blood Flow

**DOI:** 10.3389/fbioe.2021.774954

**Published:** 2021-12-07

**Authors:** Hojin Ha, Hyung Kyu Huh, Kyung Jin Park, Petter Dyverfeldt, Tino Ebbers, Dae-Hee Kim, Dong Hyun Yang

**Affiliations:** ^1^ Department of Mechanical and Biomedical Engineering, Kangwon National University, Chuncheon, South Korea; ^2^ Daegu-Gyeongbuk Medical Innovation Foundation, Medical Device Development Center, Daegu, South Korea; ^3^ Department of Electrical and Electronic Engineering, Yonsei Univeristy, Seoul, South Korea; ^4^ Department of Radiology, Asan Medical Center, University of Ulsan College of Medicine, Seoul, South Korea; ^5^ Department of Health, Medicine and Caring Science, Linköping University, Linköping, Sweden; ^6^ Center for Medical Image Science and Visualization (CMIV), Linköping University, Linköping, Sweden; ^7^ Department of Cardiology, Asan Medical Center, University of Ulsan College of Medicine, Seoul, South Korea

**Keywords:** magnetic resonace imaging, turbulence measurement, turbulent kinetic energy, turbulence production, hemodynamics

## Abstract

Imaging hemodynamics play an important role in the diagnosis of abnormal blood flow due to vascular and valvular diseases as well as in monitoring the recovery of normal blood flow after surgical or interventional treatment. Recently, characterization of turbulent blood flow using 4D flow magnetic resonance imaging (MRI) has been demonstrated by utilizing the changes in signal magnitude depending on intravoxel spin distribution. The imaging sequence was extended with a six-directional icosahedral (ICOSA6) flow-encoding to characterize all elements of the Reynolds stress tensor (RST) in turbulent blood flow. In the present study, we aimed to demonstrate the feasibility of full RST analysis using ICOSA6 4D flow MRI under physiological conditions. First, the turbulence analysis was performed through *in vitro* experiments with a physiological pulsatile flow condition. Second, a total of 12 normal subjects and one patient with severe aortic stenosis were analyzed using the same sequence. The *in-vitro* study showed that total turbulent kinetic energy (TKE) was less affected by the signal-to-noise ratio (SNR), however, maximum principal turbulence shear stress (MPTSS) and total turbulence production (TP) had a noise-induced bias. Smaller degree of the bias was observed for TP compared to MPTSS. *In-vivo* study showed that the subject-variability on turbulence quantification was relatively low for the consistent scan protocol. The *in vivo* demonstration of the stenosis patient showed that the turbulence analysis could clearly distinguish the difference in all turbulence parameters as they were at least an order of magnitude larger than those from the normal subjects.

## Introduction

Imaging hemodynamics plays an important role in the diagnosis of abnormal blood flow due to vascular and valvular diseases and monitoring the recovery of normal blood flow after surgical or interventional treatment (Ragosta, 2017; Members et al., 2021). Non-invasive measurement of hemodynamic parameters, such as velocity, pressure loss, and perfusion, has been an important marker for the management and therapy of patients with vascular diseases (Ragosta, 2017; Members et al., 2021).

While echocardiography is still a dominant imaging tool for assessing hemodynamics in clinics, volume acquisition of phase-contrast magnetic resonance imaging, also termed 4D flow MRI, is an emerging technique to characterize multi-dimensional features of hemodynamics (Caruthers et al., 2003; Falahatpisheh et al., 2016; Donati et al., 2017). 4D flow MRI quantifies not only the velocity and flow rate, but also provides various dynamic and kinematic properties of the blood flow, such as wall shear stress (WSS) (Barker et al., 2012; Bissell et al., 2013), turbulent kinetic energy (TKE) (Dyverfeldt et al., 2008; Dyverfeldt et al., 2009a; Dyverfeldt et al., 2013), vorticity (Kim et al., 2015; von Spiczak et al., 2015), pressure gradient (Ebbers et al., 2001; Krittian et al., 2012; Donati et al., 2015), and pulse wave velocity (PWV) (Markl et al., 2010; Markl et al., 2012). In addition, numerous applications of 4D flow MRI for different cardiac and vascular diseases have been introduced, and its clinical implications beyond conventional echocardiography or other diagnostic tools have been successfully demonstrated (Stankovic et al., 2014; Ha et al., 2016d; Soulat et al., 2020; Rizk, 2021).

Characterization of turbulent blood flow in the circulation system has received attention from researchers as it provides additional insights into the extent of spatiotemporal velocity fluctuation and the corresponding stress and energy. The development of turbulent flow dissipates kinetic energy into internal energy by viscous shear stress, which elevates the energy and pressure loss accordingly (Pope, 2001). The elevated viscous shear stress due to the stochastic velocity fluctuation also increases damage to the blood components, promoting hemolysis and thrombosis (Mustard et al., 1962; Smith et al., 1972; Sallam and Hwang, 1983; Lu et al., 2001; Yen et al., 2014). As the mechanical stimuli of turbulent flow are detected and transduced into endothelial cells, the pathophysiology of turbulence on the progression of atherosclerosis and vascular remodeling has also been investigated (Davies et al., 1986; Davies, 1989; Prado et al., 2006).

Although turbulence measurement using medical instruments is still challenging, there has been continuing research on turbulence quantification for developing novel hemodynamic markers. Previously, a catheter-based hot-film anemometer was used to quantify the turbulence level in the aortic flow. The turbulent intensity, frequency, and energy density of the normal and stenotic flows were, thus, successfully analyzed (Stein and Sabbah, 1976; Yamaguchi et al., 1983; Hanai et al., 1991). Since the catheter-based method is currently limited due to its invasiveness, turbulence characterization using non-invasive 4D flow MRI has been widely carried out (Dyverfeldt et al., 2006; Dyverfeldt et al., 2008; Dyverfeldt et al., 2009a; Dyverfeldt et al., 2013).

Conventional velocity measurement from the phase image of 4D flow MRI acquisition does not include turbulent flow features. The MRI sequence fills k-space data from multiple cardiac cycles. The reconstructed velocity field inherently is an ensemble average of many repeated signal acquisitions. As the reconstruction of the MRI signal using a discrete inverse Fourier transform gives the representative value of the whole spin signals within the voxel, the voxel data are also spatially averaged (Brown et al., 2014).

Recently, the application of 4D flow MRI for turbulence estimation has been widely demonstrated by utilizing the changes in MRI signal magnitude depending on intravoxel spin distribution (Dyverfeldt et al., 2006; Dyverfeldt et al., 2008; Dyverfeldt et al., 2009a; Dyverfeldt et al., 2013). Previously, TKE, which is the trace of the Reynolds stress tensor (RST), was estimated using the conventional 4D flow MRI sequence for the non-invasive measurement of turbulence in the aortic blood flow (Dyverfeldt et al., 2008). This 4D flow MRI sequence was further extended with a six-directional icosahedral (ICOSA6) flow-encoding scheme to measure all elements of RST, rather than only three diagonal elements, in turbulent flows (Ha et al., 2016e; Ha et al., 2017b; Haraldsson et al., 2018; Ha et al., 2019). Recently, it was found that multi-point flow encoding with a highly under-sampled acquisition successfully quantified the turbulence within ten minutes of scanning (Walheim et al., 2019).

Although preliminary studies on the quantification of full RST using extended 4D flow MRI have demonstrated its potential in medicine, the practical feasibility of turbulence analysis under physiological conditions has rarely been demonstrated. Most *in vitro* demonstrations have used the steady flow condition to optimize the experimental environments, such as signal-to-noise ratio (SNR) and scan time. A previous study demonstrating multi-point measurement for full RST analysis in two normal subjects and patients with valvular diseases has been the only *in vivo* study performed till date (Walheim et al., 2019). Therefore, questions still remain to be answered, for example; whether the turbulence analysis provides robust results and what happens if parameter dependency arises in cases for highly pulsatile flows, particularly for *in vivo* scan conditions.

This study aimed to investigate the performance of full RST analysis using ICOSA6 4D flow MRI under physiological conditions. First, we confirmed the feasibility of the turbulence analysis at different velocity encoding (Venc) conditions using *in vitro* experiments with a pulsatile flow condition. Second, a total of 12 normal subjects and one patient with aortic stenosis were scanned with the same sequence. The extent of the turbulence parameters from *in vivo* measurements was analyzed accordingly.

## Materials and Methods

### *In-vitro* Experimental Setup

**FIGURE 1:**
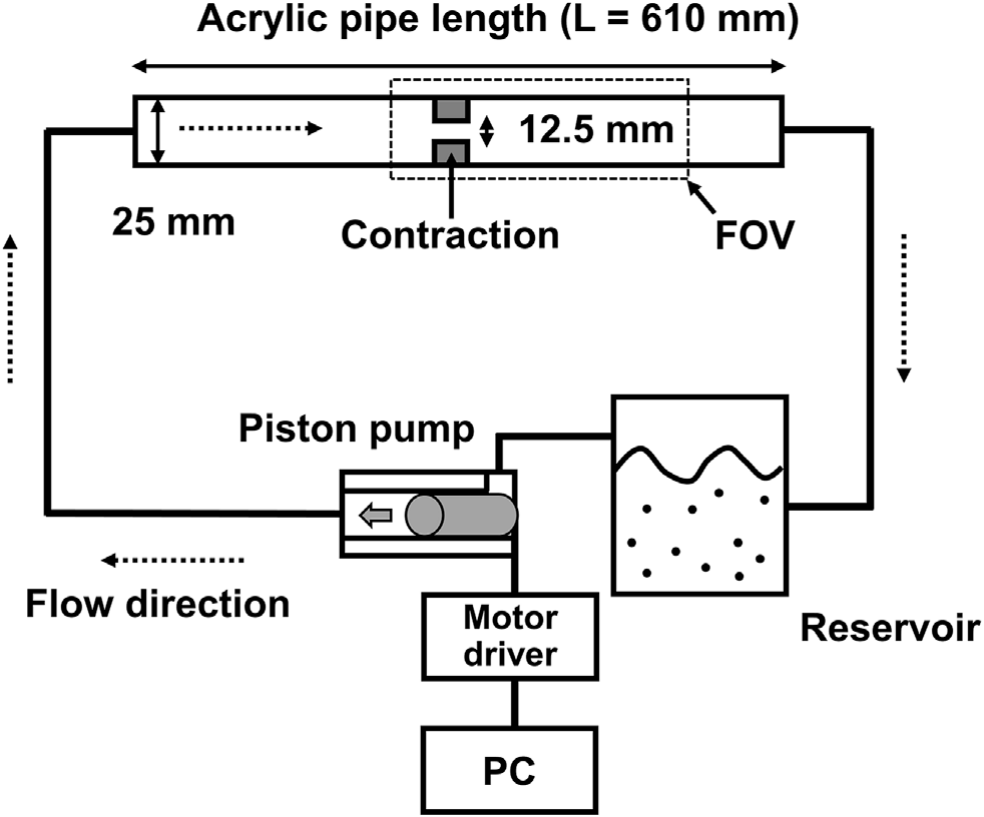
A schematic for *in-vitro* experiments.

*In vitro* measurements of 4D flow MRI were performed using an acrylic flow phantom and a cardiovascular-mimicking pulsatile flow pump (Figure 1). The stenotic phantom had a 50% reduction in length, which corresponds to a 75% reduction in area with a rectangular cross-sectional shape. The upstream and downstream diameters, without stenosis, were 25 mm. To minimize the entrance effect, 0.3 m of the straight inlet upstream of the stenosis was used to minimize the entrance effect. In addition, the same length of the outlet part was used downstream of the stenosis. The working fluids were a mixture of 60% water and 40% glycerol by mass. The density was 1,053.8 kg/m^3^, which corresponded to a dynamic viscosity of 3.72 × 10^–3^ kg/m s. The working fluid was circulated through the flow phantom with a physiological pulsatile waveform using an in-house cardiovascular pulse duplication pump (Kim et al., 2020). The in-house pulsatile pump uses a programmable piston pump to replicate human aortic blood flow waveforms at 60 beats per minute (bpm). The corresponding Womersley number $\alpha =D/2\sqrt{\rho 2\pi f/\mu }$ in the pulsatile flow was 16.7, where D is the diameter, ρ is the density, μ is the dynamic viscosity, and f is the frequency. The mean and maximum flow rates of the pulsatile flow were 3.95 L/min and 13.1 L/min, respectively. The corresponding peak Reynolds number, Re=ρuD/μ=ρQD/μA , at the inlet and stenosis regions were 2,735 and 5,471, respectively, where Q is the flow rate and A is the area (Supplementary Figure S1). The temperature of the working fluid was maintained at 20°C during the experiment to maintain the fluid properties. A 30 ml volume of MRI contrast agent (0.5 mmol/kg, gadofosveset trisodium, VasovistVR, Bayer Schering Pharma AG, Berlin, Germany) was mixed to working fluid (40 L) for better SNR during *in-vitro* measurement.

### Recruitment of Normal Subjects and Patient for In-Vivo Study

Twelve healthy volunteers and one patient with severe aortic stenosis were prospectively enrolled in this study. This study was approved by the Institutional Review Board of the Asan Medical Center (approval number: 2020-1698, Seoul, Korea). Written informed consent was obtained from all the participants. Normal subjects were confirmed to have no severe cardiovascular disease from the cardiology department before they were scanned using 4D flow MRI. One patient with severe aortic stenosis was registered for comparison with normal subjects. Echocardiography showed that the patient had a peak velocity of 4.7 m/s, which corresponds to the mean and peak pressure gradients of 53 and 89 mmHg, respectively. A demographic summary of the *in vivo* subjects is summarized in Table 1.

**TABLE 1:** Demographic characteristics of the *in-vivo* subjects.

	Case	Age (years)	Sex (F/M)	Height (cm)	Weight (kg)	BSA (m^2^)	LV EDV (mL)	LV ESV (mL)	LVEF (%)	LA diameter (mm)	Aorta (mm)
Normal	1	51	M	169.8	60.6	1.7	76	26	66	31	39
	2	59	F	161.3	61.8	1.65	70	31	56	36	27
	3	62	F	151.7	55.7	1.51	74	25	66	31	21
	4	31	M	175.7	74.7	1.9	113	41	64	29	33
	5	52	M	175.1	70.5	1.85	169	65	62	36	31
	6	67	F	151.6	61.2	1.57	102	34	67	35	30
	7	36	F	165.8	51.4	1.56	68	22	68	28	29
	8	51	F	154.4	45.6	1.41	76	29	62	26	27
	9	72	M	170.2	67.2	1.78	106	42	60	40	33
	10	56	F	160.0	60.0	1.62	102	35	66	36	30
	11	49	M	177.1	84.2	2.02	133	48	64	39	39
	12	76	F	143.9	43.5	1.31	68	27	60	28	32
Patient	1	64	M	169.4	74.2	1.85	130	46	65	37	37

BSA, body surface area; LV, left ventricle; EDV, end-diastolic volume; ESV, end-systolic volume; LVEF, left ventricular ejection fraction; LA, left atrium.

### 4D Flow MRI Measurement

The 4D flow MRI measurements for the *in vitro* experiments were as follows: A commercial 1.5T MRI scanner (1.5T Philips Achieva, Philips Medical Systems, Best, Netherlands) with a 32-channel torso coil performed the ICOSA6 sequence, which was modified to employ icosahedral flow encoding (six-directional) with a single flow-compensated reference encoding. Various velocity-encoding (Venc) parameter values (100–350 cm/s) were selected for the turbulence analysis, and 350 cm/s was used for velocity measurement. The echo time, temporal resolution, flip angle and matrix size were 2.5 ms, 3.9 ms, 10° and 128 × 128 × 25 (2.0 mm isotropic voxel), respectively. To obtain the shortest TE, a partial echo factor was set to 0.725. The total scan time for the *in vitro* study was approximately 30 min per case.

The 4D flow MRI parameters for the *in vivo* study, other than those described below, were the same as those for the *in vitro* experiments. A dStream Flex coil (Philips Medical Systems, Best, Netherlands) was used with various Venc parameters ranging from 80 to 100 cm/s for the normal subjects and 300 cm/s for the stenosis patient for turbulence quantification. TE and temporal resolution were slightly adjusted according to the scan condition, ranging from 1.9 to 2.7 ms and 3.8–4.4 ms, respectively. The matrix size range was 112–128 × 112–128 × 23–30 voxels (2.5–3.0 mm isotropic voxel). The scan time for the *in vivo* study was approximately 23 min.

### Post-processing of 4D Flow MRI Data

Raw data were exported using Pack’n Go and reconstructed offline using ReconFrame (ReconFrame, Gyrotool LLC, Zurich, Switzerland). A custom MATLAB (The MathWorks, Inc., Natick, MA) code was used to solve the linear equations to recover the velocity vector and RST, as described in previous works (Ha et al., 2017a; Ha et al., 2019). To correct the background phase errors, a no-flow velocity field (flow off) was subtracted from the *in vitro* data (Ha et al., 2019) and weighted 2nd order fitting to static tissue was used for the *in vivo* data (Ebbers et al., 2008).

**FIGURE 2:**
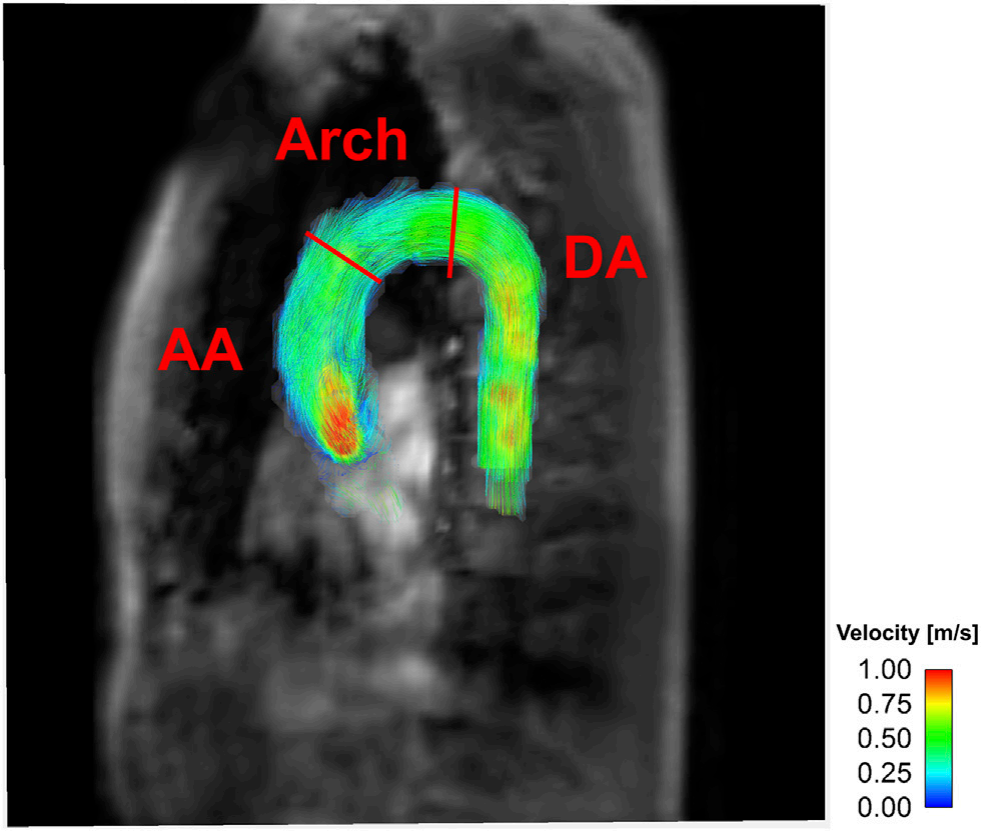
Representative velocity mapping of ICOSA6 4D flow MRI for *in-vivo*.

Magnitude and velocity images were imported into the ITK-SNAP software (v.3.8.0, University of Utah, Salt Lake City, UT) to segment the aortic flow region. The aorta was subdivided into the ascending aorta (AA), descending aorta (DA), and aortic arch (arch) by the brachiocephalic artery and the left subclavian artery (Figure 2). Aortic branches were excluded from the analysis.

### 4D Flow MRI Turbulence Quantification

The intravoxel velocity variance (IVVV) of the turbulent flow in I direction $\left(\sigma_{i}^{2}\right)$ was calculated by dividing the velocity-encoded signal magnitude S_i_(k_v_) from the reference signal magnitude S (0) and as Eq. 1 (Dyverfeldt et al., 2009b):

$$\sigma_{i}^{2}=\bar{u_{i}^{\prime }u_{i}^{\prime }}=\frac{2}{k_{v}^{2}}\ln\left(\frac{\left|S\left(0\right)\right|}{\left|S_{i}\left(k_{v}\right)\right|}\right),\left(\frac{m^{2}}{s_{2}}\right)$$ (1)

where $u_{i}^{\prime }$ denotes fluctuating velocity component and ‾ denotes an averaging operation.

**FIGURE 3:**
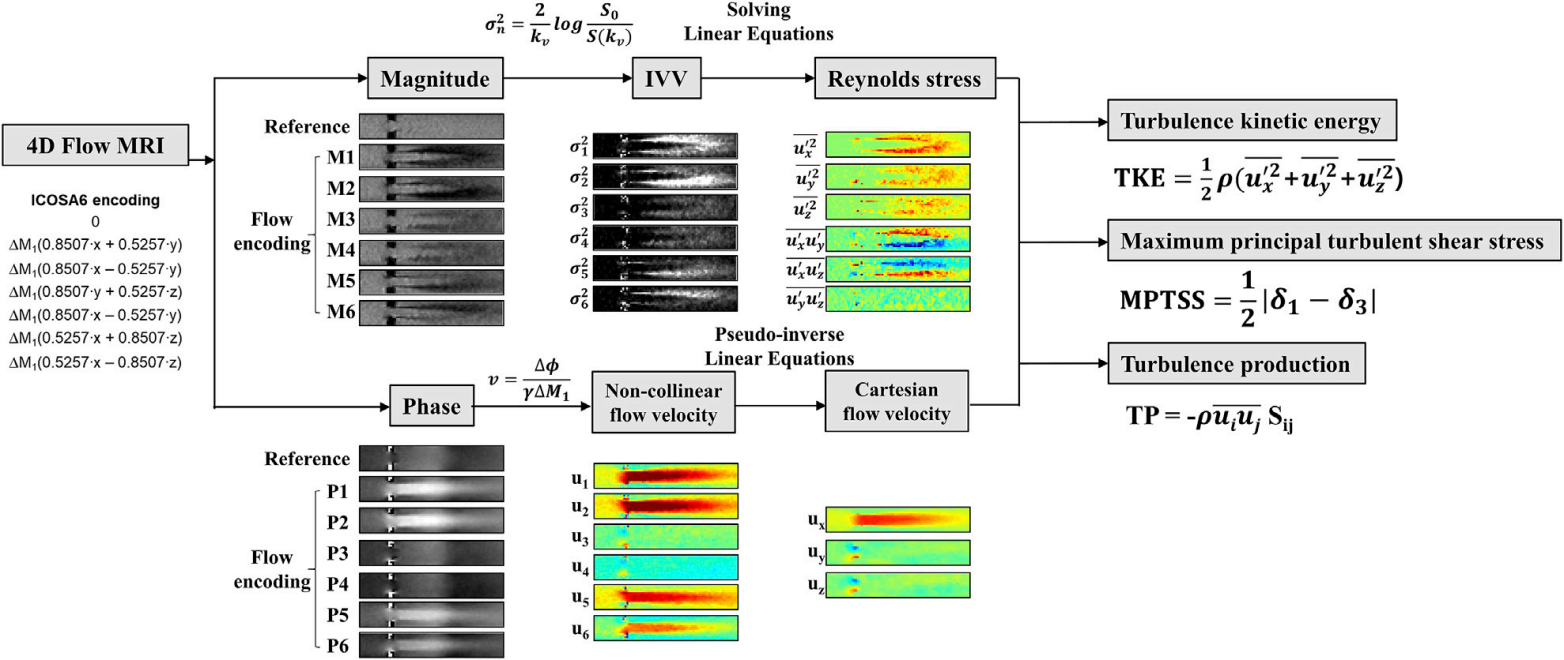
Illustration of Reynolds stress measurement and turbulence parameter analysis.

Orthogonal components (velocity vectors u, v, and w) and covariance components (Reynolds stress tensor R_ij_, a six-element symmetric tensor in Eq. 2) can be simultaneously calculated by solving linear equations from six non-orthogonal velocity encodings (Figure 3) (Haraldsson et al., 2018; Ha et al., 2019).

$$R=\;-\rho \left[\begin{array}{ccc} \bar{u_{1}^{\prime }u_{1}^{\prime }} & \bar{u_{1}^{\prime }u_{2}^{\prime }} & \bar{u_{1}^{\prime }u_{3}^{\prime }}\\ \bar{u_{2}^{\prime }u_{1}^{\prime }} & \bar{u_{2}^{\prime }u_{2}^{\prime }} & \bar{u_{2}^{\prime }u_{3}^{\prime }}\\ \bar{u_{3}^{\prime }u_{1}^{\prime }} & \bar{u_{3}^{\prime }u_{2}^{\prime }} & \bar{u_{3}^{\prime }u_{3}^{\prime }} \end{array}\right]$$ (2)

The turbulent kinetic energy TKE with in the flow can be described from the IVVV of each direction as follows:

$$TKE=\;\frac{1}{2}\rho \sum_{i=1}^{3}\sigma_{i}^{2}=\;\frac{1}{2}\rho \left(\bar{u_{1}^{\prime }u_{1}^{\prime }}+\bar{u_{2}^{\prime }u_{2}^{\prime }}+\bar{u_{3}^{\prime }u_{3}^{\prime }}\right),\left(\frac{J}{m^{3}}\right)$$ (3)

where ρ is the fluid density. The voxel-wise integration of TKE provides total TKE with units of J or mJ.

The maximum principal turbulent shear stress (MPTSS) was estimated using principal stress analysis. MPTSS can be calculated as follows:

$$MPTSS=\;\frac{1}{2}\left(\delta_{1}-\delta_{3}\right),\left(\text{Pa}\right)$$ (4)

where the δ is the eigenvalues of RST $\left(\delta_{1}>\delta_{2}>\delta_{3}\right)$ .

Turbulent production (TP) can directly be computed as follows (Ha et al., 2017a; Ha et al., 2019):

$$TP=R_{ij}S_{ij},\left(\frac{W}{m^{3}}\right)$$ (5)

Here, S_ij_ denotes the strain rate tensor of the velocity field. Voxel-wise integration of TP and multiplying the density provides a total TP with a unit of W or mW.

The turbulence parameters near the luminal surface were estimated separately to estimate the impact of turbulence on the vessel wall. Near-wall TKE (nwTKE), near-wall MPTSS (nwMPTSS), and near-wall TP (nwTP) were calculated as previously described (Ziegler et al., 2017). In short, for near-wall estimation, the number of turbulence parameters near the luminal surface was obtained using a convolution kernel with a 3 × 3 mean filter.

### Statistics

A Shapiro–Wilk test was performed to check the normality of the data. The parametric data were described as mean ± standard deviation, while non-parametric data were described as median (1st quartile, 3rd quartile) throughout the manuscript.

## Results

### *In-vitro* Turbulence Quantification Under Pulsatile Flow

**FIGURE 4:**
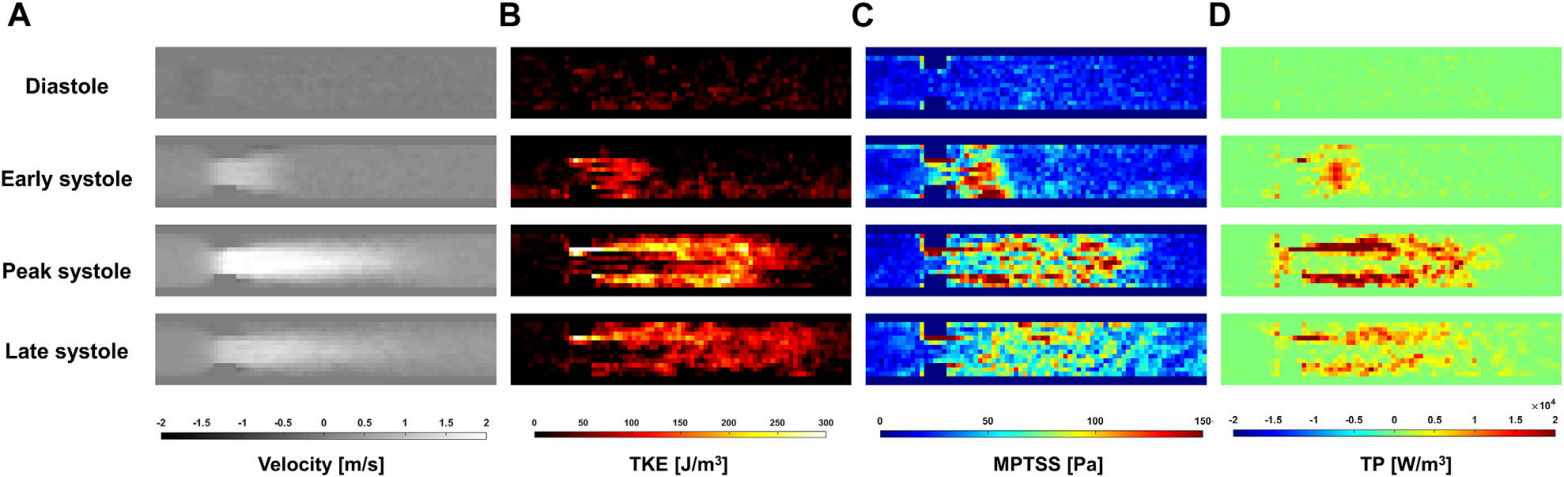
Temporal variation of **(A)** Velocity, **(B)** turbulent kinetic energy (TKE), **(C)** maximum principal turbulent shear stress (MPTSS), **(D)** turbulence production (TP) through the stenosis at Venc of 200 cm/s.

**FIGURE 5:**
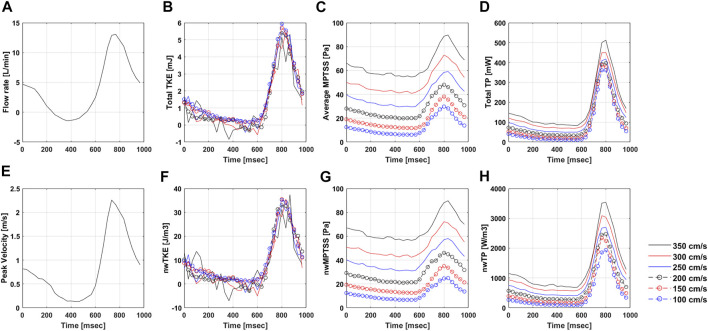
Temporal variation of **(A)** Flow rate, **(B)** total TKE, **(C)** Average MPTSS, **(D)** total TP, **(E)** Peak velocity, **(F)** nwTKE, **(G)** nwMPTSS, and **(H)** nwTP at different Venc parameters. Note that the flow rate and peak velocity are only measured at Venc of 350 cm/s.

The ICOSA6 4D Flow MRI successfully visualized the pulsatile flow waveform that generated a strong jet flow through the stenosis (Figure 4). These turbulence parameters exhibited the highest values around the boundary layer of the jet flow. Turbulence parameters (TKE, MPTSS, and TP) started to increase during the early systole phase and reached a maximum at the peak systole phase of the cycle (Figures 4, 5). The mean and peak velocity during the pulsatile cycle were 0.83 m/s and 2.26 m/s and corresponding flow rates were 3.95 L/min and 13.1 L/min, respectively.

**FIGURE 6:**
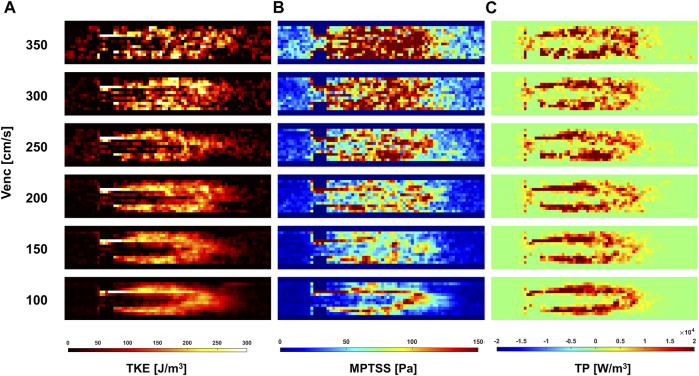
Effect of Venc on **(A)** TKE, **(B)** MPTSS, **(C)** TP at peak systole.

The quality of turbulence quantification was dependent on the Venc parameter, which determines the SNR of the measurement (Figures 5, 6). The effect of the Venc-dependent SNR on turbulence quantification varied with the turbulence parameters. The measurement with a higher Venc resulted in a higher noise level in TKE (Figures 5, 6A). The maximum difference due to Venc was 26.9 and 10.3% for the mean and maximum total TKE, respectively (Table 2). In contrast, a higher Venc resulted in a noise-induced bias in the MPTSS and TP (Figures 6B,C). Mean and maximum MPTSS at Venc = 350 cm/s were 5.1 and 3.0 folds larger than those at Venc = 100 cm/s. Mean and maximum total TP at Venc = 350 cm/s were 2.4 and 1.4 folds larger than those at Venc = 100 cm/s. The near-wall turbulence parameters exhibited similar behaviors (Table 2).

**TABLE 2:** Summary of turbulence parameters from the *in-vitro* experiments.

Venc (cm/s)	Total TKE (mJ)		Average MPTSS (Pa)		Total TP (mW)		nwTKE (J/m^3^)		nwMPTSS (Pa)		nwTP (kW/m^3^)	
	Mean	Max	Mean	Max	Mean	Max	Mean	Max	Mean	Max	Mean	Max
350	1.2	5.3	64.7	89.8	177.6	512.5	7.9	37.4	66.1	89.7	1.4	3.5
300	1.2	5.4	50.5	72.9	154.8	450.0	7.5	35.6	51.7	72.2	1.2	3.1
250	1.4	5.4	38.7	59.1	128.7	417.7	8.2	33.3	39.7	58.1	0.9	2.7
200	1.3	5.4	28.4	48.0	106.9	397.6	8.4	32.9	29.2	46.1	0.8	2.5
150	1.5	5.7	19.1	38.0	89.5	387.2	9.5	35.5	19.1	34.9	0.6	2.3
100	1.6	5.9	12.8	29.7	73.2	361.7	9.9	35.2	12.2	24.9	0.5	1.9

TKE, turbulent kinetic energy; MPTSS, maximum principal turbulence shear stress; TP, turbulence production; nw, near-wall.

### *In-vivo* Turbulence Analysis

**FIGURE 7:**
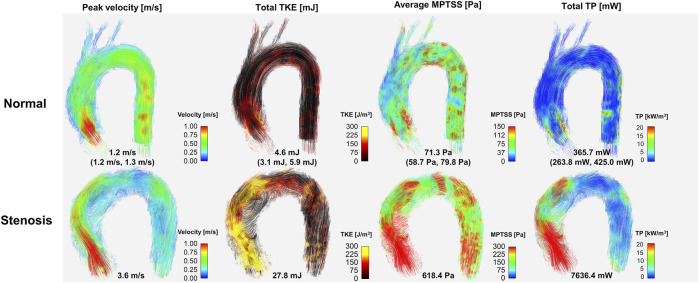
Comparison hemodynamics in normal subjects and patient with severe aortic stenosis. Note that representative normal subject (case #1) was used for color mapping. Values for the normal subject are median (1st quartile, 3rd quartile) of the data. Velocity, TKE, MPTSS and TP for all normal subjects are also shown in the Supplementary Figures S2–S5.

Twelve normal volunteers were scanned with the ICOSA6 sequence to perform flow and turbulence quantification. The blood flow through the aortic valve developed a high-velocity jet flow in the ascending aorta (Figure 7). TKE, MPTSS, and TP mapping at the peak systole phase clearly visualized the local development of turbulence with a reasonable SNR (Supplementary Figures S2–S5).

**FIGURE 8:**
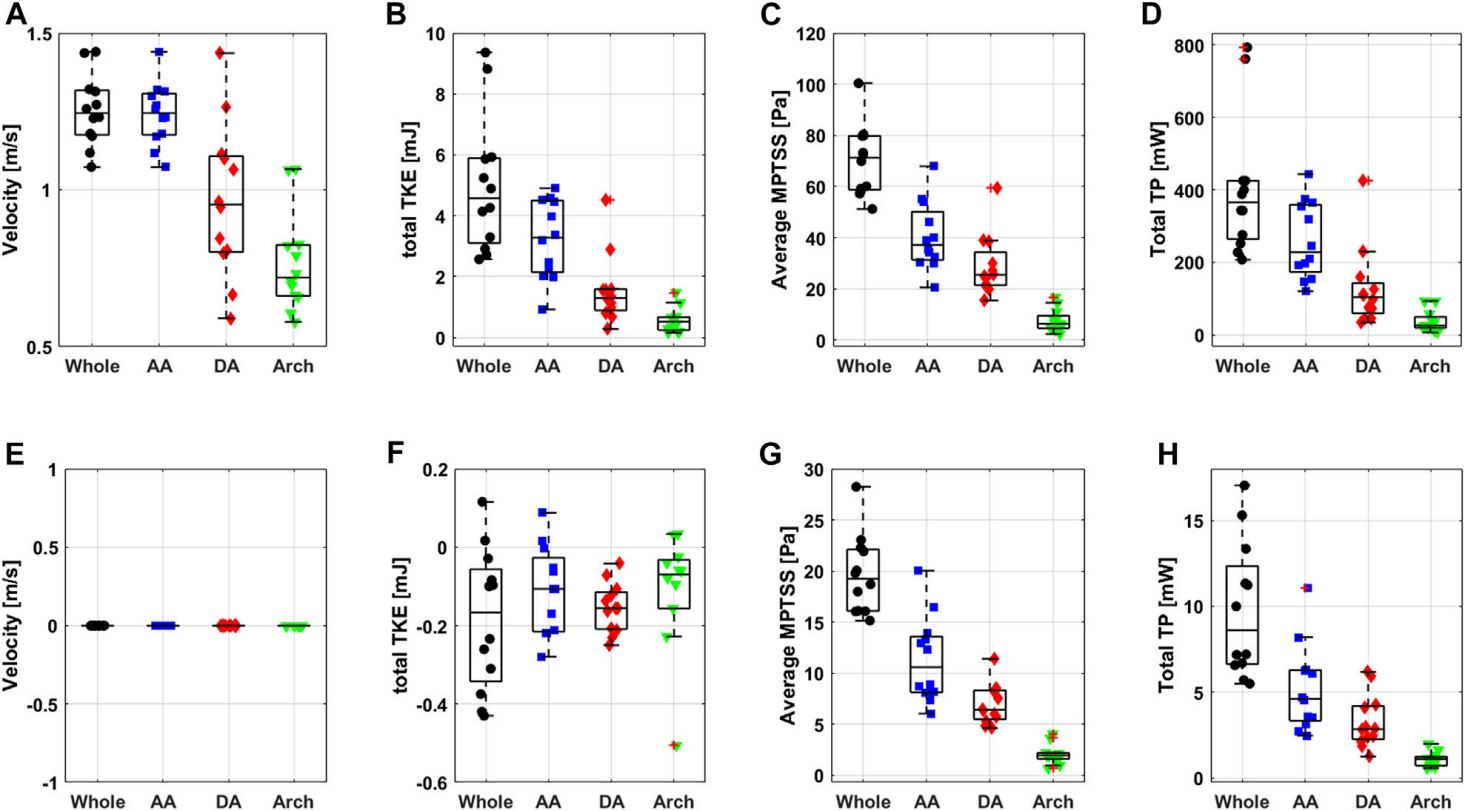
Boxplot of peak systolic **(A)** velocity, **(B)** total TKE, **(C)** average MPTSS, **(D)** total TP and diastolic **(E)** velocity, **(F)** total TKE, **(G)** average MPTSS and **(H)** total TP.

Most of the hemodynamic parameters of the normal subjects were within the confined range (Figure 8). The peak velocities of the normal subjects were 1.2 m/s (1.2 m/s, 1.3 m/s). Data are shown as median (1st quartile, 3rd quartile). The total TKE, MPTSS and TP of the normal subjects at the peak systole were 4.6 mJ (3.1 mJ, 5.9 mJ), 71.3 Pa (58.7 Pa, 79.8 Pa), 365.7 mW (263.8 mW, 425.0 mW), respectively. Among them, most of the turbulence was focused on the ascending aorta.

The turbulence parameters at the diastolic phase were significantly smaller than those at the peak systolic phase. Total TKE and total TP were at least an order of magnitude smaller than those at the peak systolic phase. The average MPTSS was approximately one-third of that at the peak systole phase (Figure 8 and Table 3). The total TKE, MPTSS and total TP of the normal subjects at the diastolic phase were −0.2 mJ (−0.3 mJ, −0.1 mJ), 19.3 Pa (16.1 Pa, 22.1 Pa), 8.6 mW (6.6 mW, 12.3 mW), respectively. While the total TKE values at the diastolic phase were almost negligible regardless of the vascular region, almost half of the MPTSS and total TP developed at the ascending aorta.

**TABLE 3:** Summary of turbulence parameters from the *in-vivo* normal subjects.

	Total TKE (mJ)			
	Whole	AA	DA	Arch
Peak Systole	4.6 (3.1, 5.9)	3.3 (2.1, 4.5)	1.3 (0.9, 1.6)	0.5 (0.2, 0.7)
Diastole	−0.2 (−0.3, −0.1)	−0.1 (−0.2, 0.0)	−0.2 (−0.2, −0.1)	−0.1 (−0.2, 0.0)
	**Average MPTSS (Pa)**			
	Whole	AA	DA	Arch
Peak Systole	71.3 (58.7, 79.8)	37.1 (31.3, 50.1)	25.4 (21.4, 34.2)	6.4 (4.6, 9.4)
Diastole	19.3 (16.1, 22.1)	10.6 (8.1, 13.6)	6.4 (5.5, 8.3)	1.9 (1.6, 2.2)
	**total TP (mW)**			
	Whole	AA	DA	Arch
Peak Systole	365.7 (263.8, 425.0)	227.6 (173.2, 358.9)	104.4 (19.7, 49.7)	25.9 (19.7, 49.7)
Diastole	8.6 (6.6, 12.3)	4.6 (3.3, 6.3)	2.8 (2.3, 4.2)	1.1 (0.7, 1.2)
	**nwTKE (J/m^3^)**			
	Whole	AA	DA	Arch
Peak Systole	44.1 (34.8, 57.4)	69.0 (47.7, 78.2)	36.1 (28.1, 47.1)	29.2 (24.3, 40.3)
Diastole	−2.0 (−4.0, −1.4)	−2.8 (−4.7, −0.7)	−4.3 (−6.1, −2.6)	−4.9 (−13.9, −2.8)
	**nwMPTSS (Pa)**			
	Whole	AA	DA	Arch
Peak Systole	72.1 (61.2, 80.9)	80.4 (72.2, 94.4)	73.0 (64.8, 88.1)	51.2 (38.4, 69.2)
Diastole	19.2 (16.1, 22.0)	20.1 (18.0, 23.8)	16.4 (14.6, 18.8)	15.0 (13.7, 18.2)
	**nwTP (W/m^3^)**			
	Whole	AA	DA	Arch
Peak Systole	3,620.8 (3,160.1, 4,627.6)	5,634.0 (4,175.9, 6,771.9)	3,173.0 (2092.3, 3,788.3)	2,024.7 (1370.2, 3,128.6)
Diastole	93.4 (80.6, 127.0)	102.9 (85.6, 138.6)	85.7 (63.8, 103.7)	76.6 (61.6, 85.1)

TKE, turbulent kinetic energy; MPTSS, maximum principal turbulence shear stress; TP, turbulence production; nw, near-wall; AA, ascending aorta; DA, descending aorta. Data are shown as median (1st quartile, 3rd quartile).

The near-wall turbulence parameters were the largest in the ascending aorta (Table3 and Supplementary Figure S6). The nwTKE, nwMPTSS and nwTP of the ascending aorta at the peak systole were 69.0 J/m^3^ (47.7 J/m^3^, 78.2 J/m^3^), 80.4 Pa (72.2 Pa, 94.4 Pa), 5,634.0 W/m^3^ (4,175.9 W/m^3^, 6,771.9 W/m^3^), respectively, while those of the whole aorta were 44.1 J/m^3^ (34.8 J/m^3^, 57.4 J/m^3^), 72.1 Pa (61.2 Pa, 80.9 Pa), 3,620.8 W/m^3^ (3,160.1 W/m^3^, 4,627.6 W/m^3^), respectively. Diastolic nwTKE and nwTP were at least an order of magnitude smaller than those at the peak systolic phase. The nwMPTSS was approximately one-third of that at the peak systole phase (Table 3).

The *in vivo* demonstration of ICOSA6 turbulence quantification for a stenosis patient with an aortic velocity of 3.6 m/s showed that all turbulence parameters were at least an order of magnitude larger (Figure 7). The total TKE, MPTSS, and total TP of the patient at the peak systole were 27.8, 618.4 Pa and 7,636.4 mW, respectively.

## Discussion

This study focuses on demonstrating the performance of full RST analysis using ICOSA6 4D flow MRI under physiological conditions. The key results of the study are as follows:

1) Turbulence quantification from *in vitro* pulsatile flow experiments can be affected by the SNR of the measurement. The effect of the Venc-dependent SNR on turbulence quantification varied with the turbulence parameters. While total TKE was less affected, MPTSS and TP had a noise-induced bias.
2) An *in vivo* study of normal subjects showed that most of the hemodynamic parameters were within the confined range. The impact of the subject-variability on turbulence quantification was relatively low for the consistent scan protocol.
3) The *in vivo* demonstration of the stenosis patient showed that the turbulence analysis could clearly distinguish the differences of all turbulence parameters as they were at least an order of magnitude larger than those from the normal subjects. The discrepancy between the normal and patient was much larger than the effect of SNR.

Validation of novel hemodynamic parameters under various conditions is crucial for the transition of a new biomarker from research to clinical routine. Since the TKE estimation using 4D flow MRI was demonstrated at the *in vitro* stenotic flow phantom (Dyverfeldt et al., 2006), various subsequent experiments confirmed the feasibility of the method under various measurement conditions (Dyverfeldt et al., 2006; Ha et al., 2016a; Petersson et al., 2016; Ziegler et al., 2017). Based on the results of *in vitro* experiments, TKE has been widely investigated as a clinical biomarker (Dyverfeldt et al., 2013; Zajac et al., 2015; Fredriksson et al., 2018; Ha et al., 2018). In contrast to TKE, other turbulence parameters from the RST have not yet been investigated. Since full RST measurements were demonstrated (Haraldsson et al., 2018), the following studies have attempted to study the accuracy and robustness of the RST measurement under limited steady flow conditions (Ha et al., 2020; Kim et al., 2021). The present study strengthens the feasibility of full RST analysis by adding the results under physiological pulsatile flow conditions. It is noted that the sample size for the *in vivo* normal study was small. We added one patient data set to show that the degree of turbulence in the patient is at least an order of magnitude higher than in the normal subjects. Elevated turbulence level in patients with valvular and vascular disease has also been reported previously (Dyverfeldt et al., 2008; Dyverfeldt et al., 2013). Adding a few more patients would not affect the results of the present study. Successful demonstration of RST analysis for a small group of *in vivo* studies will be an important bridge for upcoming large clinical trials.

The turbulence parameters from the full RST characterize different clinical aspects of turbulent flow. TKE is the kinetic energy associated with eddies in turbulent flow. Physically, TKE is a measure of how much turbulent energy is currently developed due to vascular coactation or valvular stenosis. The MPTSS indicates the extent to which shear stress is developed due to turbulence. Elevation of turbulent shear stress on the vessel wall or blood components can describe the risk of hemolysis (Grigioni et al., 1999). TP indicates how much TKE is produced, which will eventually dissipate. This indicates how much energy is taken from the mean flow to produce turbulence, and how much energy is dissipated into internal energy such as heat (Tennekes and Lumley, 1972). The TP has been investigated to indicate the irreversible pressure loss due to turbulence (Ha et al., 2019). Although conventional 4D flow MRI can also measure TKE, MPTSS and TP, it can only be estimated with full elements of the RST. The ICOSA6 4D flow MRI used in this study provides all turbulence parameters in compensation for three additional flow encodings and corresponding scan times.

The *in vitro* demonstration shows that the effect of the SNR on the turbulence quantification differs between the turbulence parameters. The Venc-dependent SNR adds the Gaussian noise distribution on TKE unless too much turbulence causes the flow-encoded signal magnitude to be less than the noise level (Dyverfeldt et al., 2009b; Ha et al., 2016a). Therefore, the choice of Venc affects the uncertainty of the TKE, but not the accuracy. The TKE results from the present study agree with those of previous studies. Compared to the measurement at the lowest Venc, higher Venc measurements showed larger noise-induced fluctuations (Figure 5). In contrast to TKE, MPTSS largely varied with the SNR. MPTSS is estimated from the eigenvalues of the RST, which are the solutions of the characteristic equation (Fung, 1977). The coefficients of the characteristic equation are obtained from the summation and multiplication of the RST elements. Therefore, the Gaussian noise distribution on the elements of the RST does not produce the same noise distribution on the MPTSS. When the MPTSS is expressed with the principal stress, it includes the square root of the principal stress squared. Therefore, a higher noise level increases the MPTSS, as shown in Figures 5, 6. The overestimation of MPTSS was also described in a previous study using Monte Carlo simulation (Walheim et al., 2019). While it was less obvious than MPTSS, TP also showed a Venc-dependent bias. The mean and maximum MPTSS at Venc = 350 cm/s were 5.1 and 3.0 folds larger than those at Venc = 100 cm/s, and the mean and maximum total TP were 2.4 and 1.4 folds larger at the same conditions. Considering that the clinical protocol for turbulence quantification using 4D flow MRI usually uses the same or similar parameters for all cohorts, such large discrepancies due to Venc-dependent SNR changes will only be shown in the worst-case scenario (Dyverfeldt et al., 2008; Dyverfeldt et al., 2013).

The subject-variability including subject-dependent SNR-variability played a minor role in turbulence quantification in the *in vivo* study. Hemodynamic parameters for the normal subjects were relatively similar despite a wide spectrum of age and corresponding height, weight, and cardiovascular indices (Figure 5 and Table 1). This was mostly because consistent scan parameters were used throughout the *in vivo* study. Venc between 80 cm/s to 100 cm/s and the voxel resolution between 2.5 and 3.0 mm were used for the normal subjects. Despite the *in vitro* experiments on steady flow, a previous study also showed that the turbulence quantification changes less than 11.5% for TKE and 33.9% for TP when the practical range of Venc between 100 cm/s and 200 cm/s was used (Ha et al., 2020). Walheim et al. also analyzed the effect of the SNR on the turbulence parameters (Walheim et al., 2019). Monte-Carlo simulation from the study also showed that SNR played a minor role in TKE and MPTSS compared to the effect of image resolution.

The feasibility of ICOSA6 4D flow MRI for patients with aortic stenosis showed that the turbulence analysis could clearly distinguish the differences in all turbulence parameters. TKE, MPTSS, and TP were at least an order of magnitude larger than those in the normal subjects. It is noteworthy that the optimum choice of Venc for 4D flow MRI turbulence quantification is related to the extent of turbulence in the flow. The use of a very small Venc value may result in excessive turbulence-related signal loss, which can lead to the underestimation of turbulence parameters owing to the Rician noise distribution (Dyverfeldt et al., 2009a). For this reason, usually, a larger Venc for stenotic flow than that for normal aortic flow is used (Dyverfeldt et al., 2013). Therefore, the turbulence parameters for the patient can be overestimated, particularly for the MPTSS and TP. Considering that the *in vitro* study showed that maximum MPTSS and TP at Venc = 350 cm/s were 3.0 folds and 1.4 folds larger than those at Venc = 100 cm/s, the elevation of turbulence parameters in the stenosis patient observed in this study is far beyond the effect of the Venc-dependency effect. However, care should be taken when turbulence parameters from different Venc parameters are to be compared.

It should be noted that turbulence measurement using 4D flow MRI can result in unphysical values, such as negative TKE at some voxels. This phenomenon is mostly due to background noise in the magnitude image. Since, the development of turbulence increases the signal loss in the flow-encoded image, the turbulence level is quantified by determining the signal loss in the flow-encoded image compared to the reference image (Dyverfeldt et al., 2009b). When turbulence-related signal loss is relatively small because the extent of turbulence is negligible or the first moment of bipolar gradient is too small to produce intravoxel dephasing, there are some chances for some voxels of the flow-encoded image have larger intensity than those of the reference image (Ha et al., 2016a). In contrast, the signal loss at the flow-encoded image quantifies the positive IVVV; a larger intensity in the flow-coded image is interpreted as negative IVVV. In general, this noise distribution affects the voxel-wise TKE but has less effect on the total TKE because the noise cancels out during the volumetric integration (Ha et al., 2016b). Despite volumetric integration, some extent of uncertainty may still affect the results, so that total TKE becomes negative when the turbulence is almost negligible (Ha et al., 2016b). To minimize the effect of noise on turbulence quantification, multi-Venc measurements have been used to optimize the results by finding the best possible estimates (Ha et al., 2019). A recent study filtered negative diagonal components of the RST to enforce positive IVVV (Marlevi et al., 2020). Filtering based on the physically realizable states of turbulence was also considered (Andersson et al., 2021).

The increased acquisition time of ICOSA6 4D flow MRI has been an inherent drawback for clinical use. Unlike conventional four-directional encoding, this sequence employs seven flow encodings, which increase the scan time by up to 75%. However, recent developments in various acceleration techniques, including compressed sensing and local low-rank, have been successfully applied to reduce the scan time without sacrificing the critical flow information (Zhang et al., 2015; Ma et al., 2019). In addition, Walheim et al. reported that faster turbulence quantification can be performed within ten minutes using highly under-sampled 5D flow MRI acquisition with locally low-rank image reconstruction (Walheim et al., 2019). We speculate that the scan time of turbulence quantification will become trivial as acceleration techniques are further developed.

It is noted that this study does not include the validation of MRI turbulence measurements against other engineering flow measurements. However, the feasibility and validation of MRI turbulence measurements have been previously demonstrated again laser Doppler anemometer (Dyverfeldt et al., 2006), particle image velocimetry (Knobloch et al., 2014; Ha et al., 2016c), and computational fluid dynamics (Petersson et al., 2016).

One of the limitations of the present study is that the uncertainty level of each measurement has not been presented. This would require multiple measurements of the same flow conditions, which is not feasible for *in-vivo* subjects due to the long scan time. Instead, the present study investigated the same flow conditions at different Venc and SNR. In addition, a level of uncertainty for *in-vivo* measurements has been studied by observing the range of the turbulence parameters in the normal cohort.

Another limitation of the present study is that the sample size for the *in vivo* normal study was small. The current results do not represent the true normal turbulence level. Based on the successful demonstration of turbulence analysis for a small group study, this will trigger upcoming large clinical trials. The atlas of turbulence parameters at different age, sex, and disease groups will be followed in the future.

## Data Availability

The original contributions presented in the study are included in the article/Supplementary Material, further inquiries can be directed to the corresponding author.
