# Supplementary material for: In-vitro and In-Vivo Assessment of 4D Flow MRI Reynolds Stress Mapping for Pulsatile Blood Flow
Source: Front Bioeng Biotechnol. 2021 Dec 7;9:774954. doi: 10.3389/fbioe.2021.774954 (PMC8691458; doi:10.3389/fbioe.2021.774954)
Supplement: Supplementary file 1 [file DataSheet1.DOCX]

Supplementary Material

## Supplementary Figures


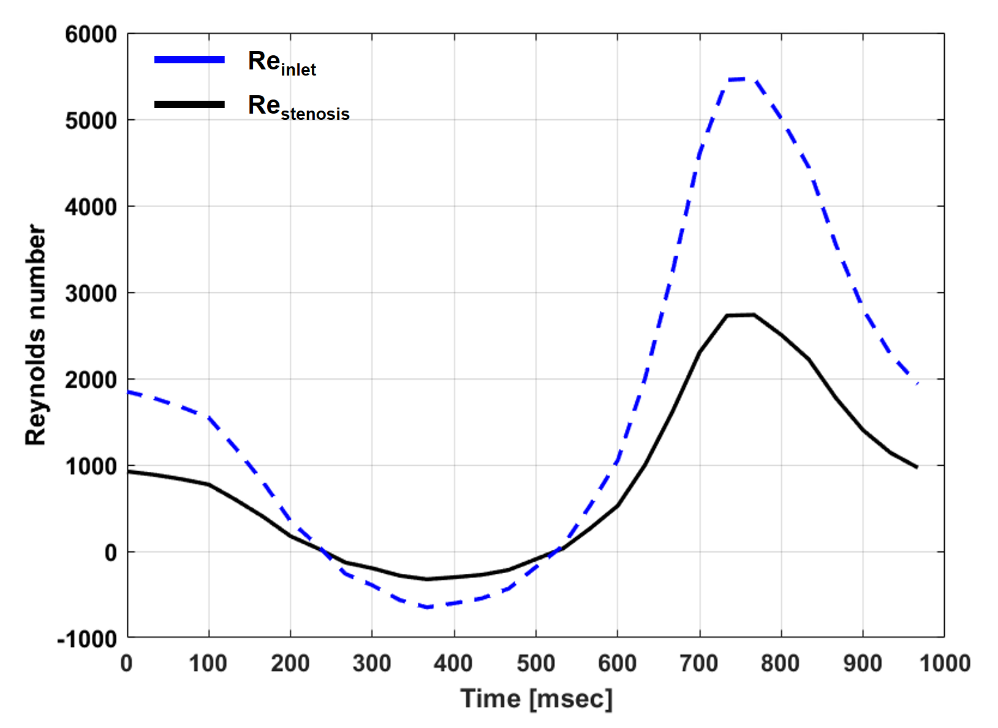


Supplementary Figure 1. Reynolds number of the pulsatile flow for in-vitro experiments. Re_inlet_ and Re_stenosis_ indicate that the Reynolds number based on the inlet diameter and stenosis diameter, respectively. Peak values of Re_inlet_ and Re_stenosis_ were 5,471 and 2,735. Note that the negative value indicates the retrograde flow.


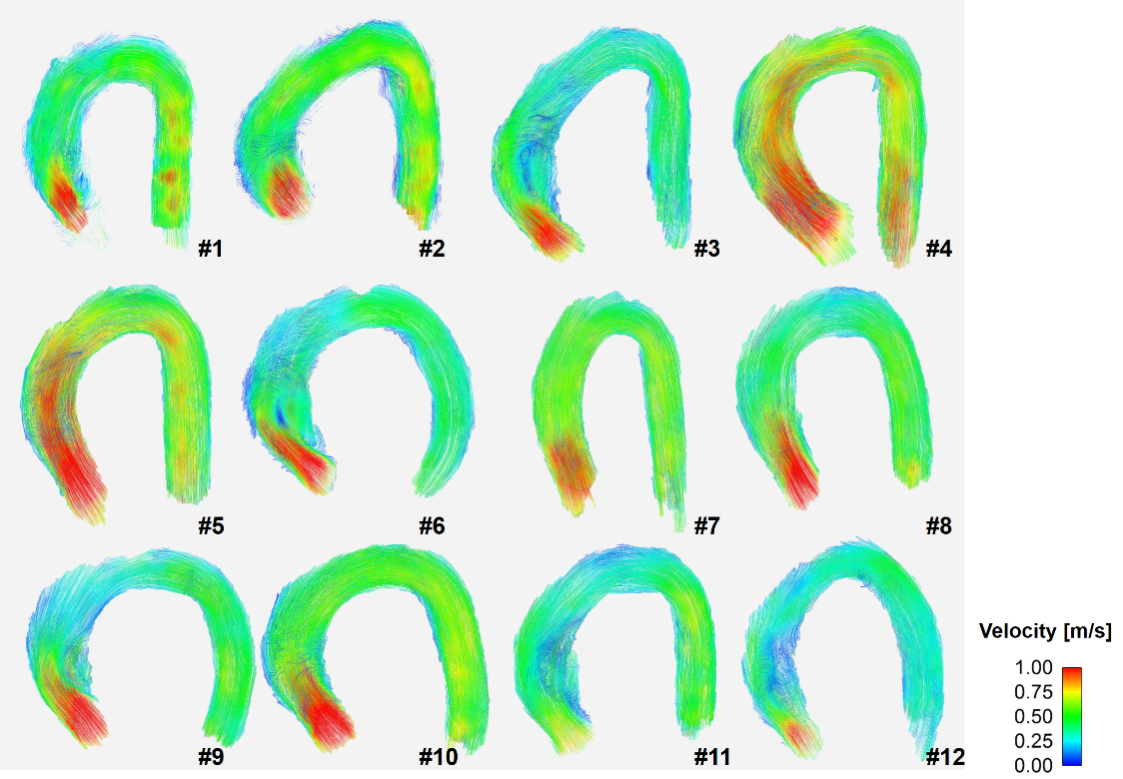


Supplementary Figure 2. Peak systolic velocity of in-vivo normal subjects.


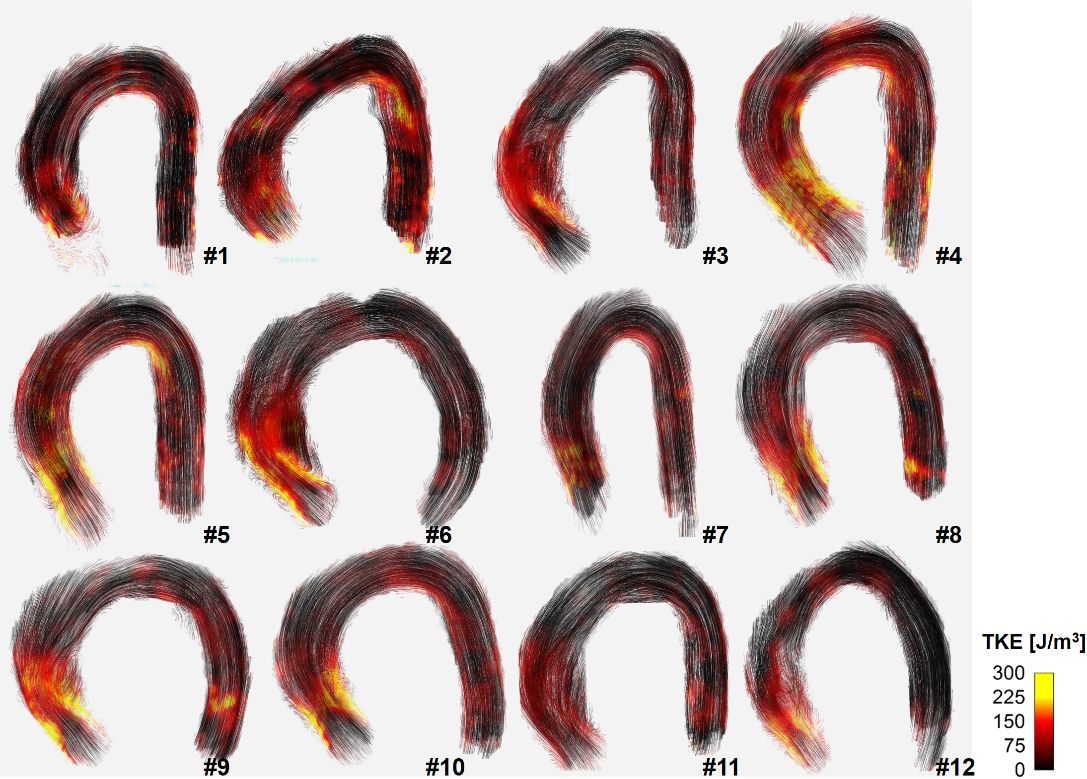


Supplementary Figure 3. Peak systolic TKE of in-vivo normal subjects.


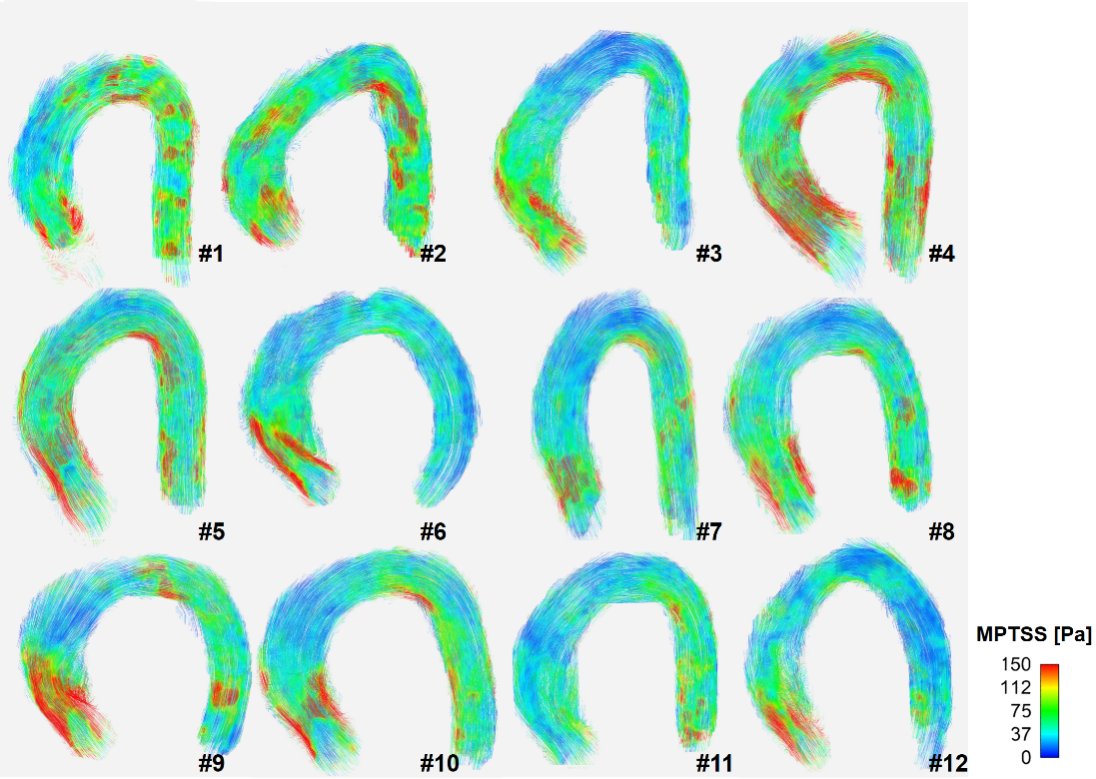


Supplementary Figure 4. Peak systolic MPTSS of in-vivo normal subjects.


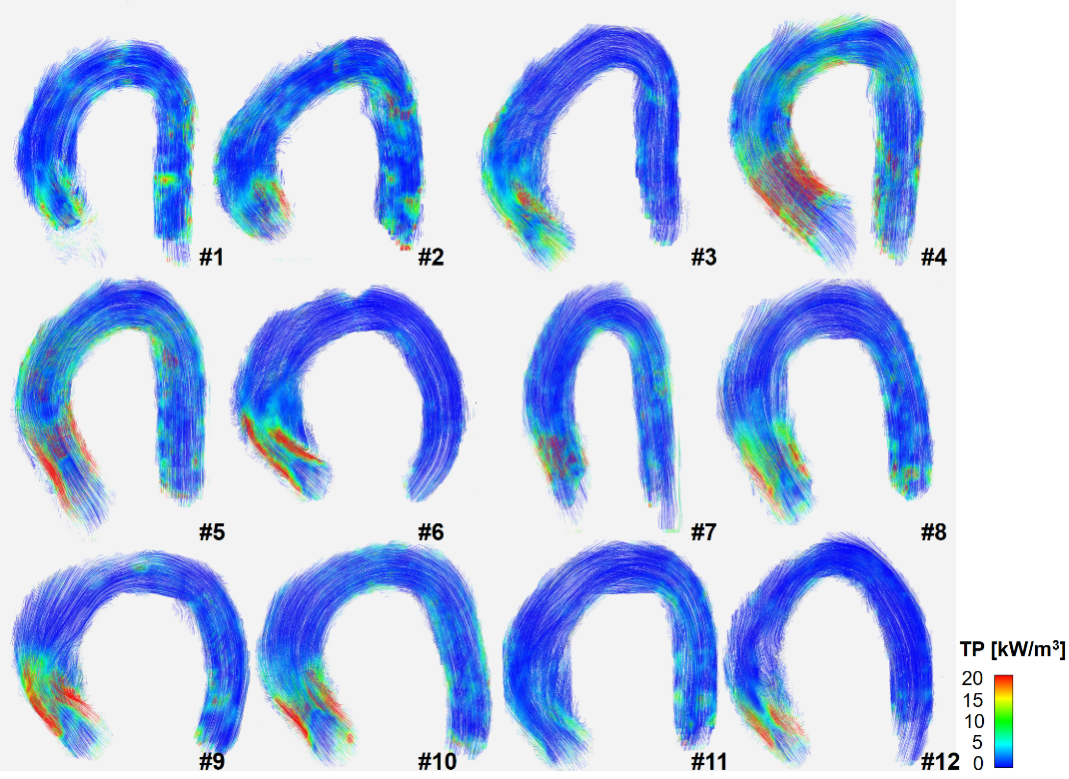


Supplementary Figure 5. Peak systolic TP of in-vivo normal subjects.

*
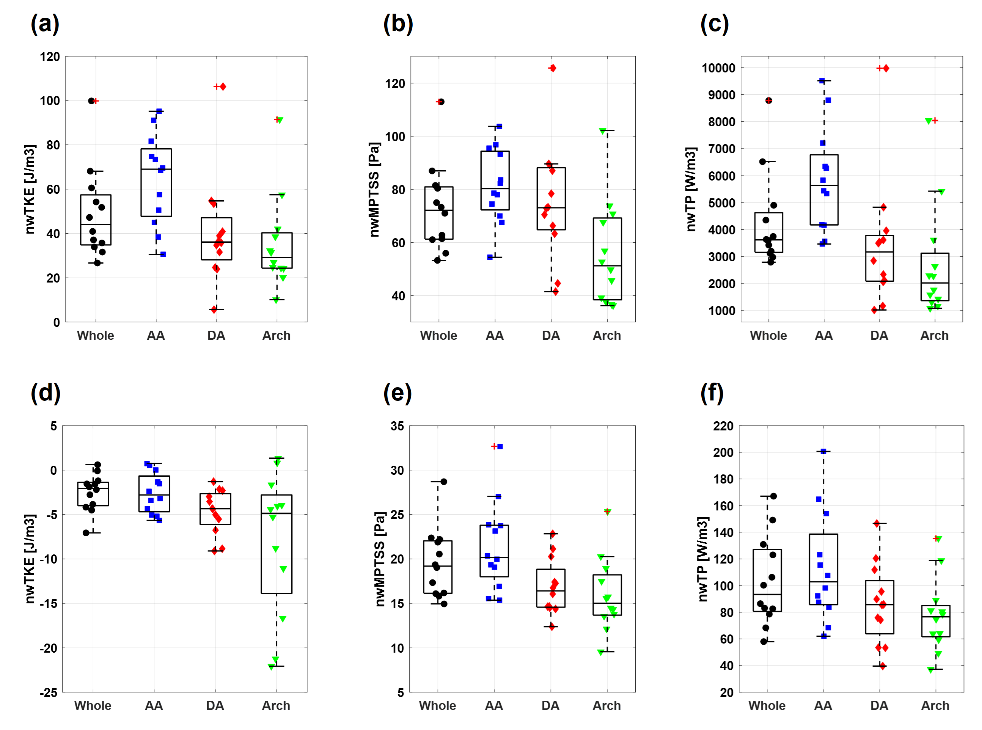
*

**Supplementary** **Figure A6**. Boxplot of peak systolic (a) nwTKE, (b) nwMPTSS, (c) nwTP and diastolic (d) nwTKE, (e) average MPTSS and (f) total TP.
